# Supplementary material for: Building molecular model series from heterogeneous CryoEM structures using Gaussian mixture models and deep neural networks
Source: bioRxiv. 2024 Sep 27:2024.09.27.615511. Preprint. [Version 1] doi: 10.1101/2024.09.27.615511 (PMC11463374; doi:10.1101/2024.09.27.615511)
Supplement: 1 [file NIHPP2024.09.27.615511V1-supplement-1.pdf]

bioRxiv preprint doi: https://doi.org/10.1101/2024.09.27.615511; this version posted September 27, 2024. The copyright holder for this preprint (which was not certified by peer review) is the author/funder, who has granted bioRxiv a license to display the preprint in perpetuity. It is made available under aCC-BY 4.0 International license.

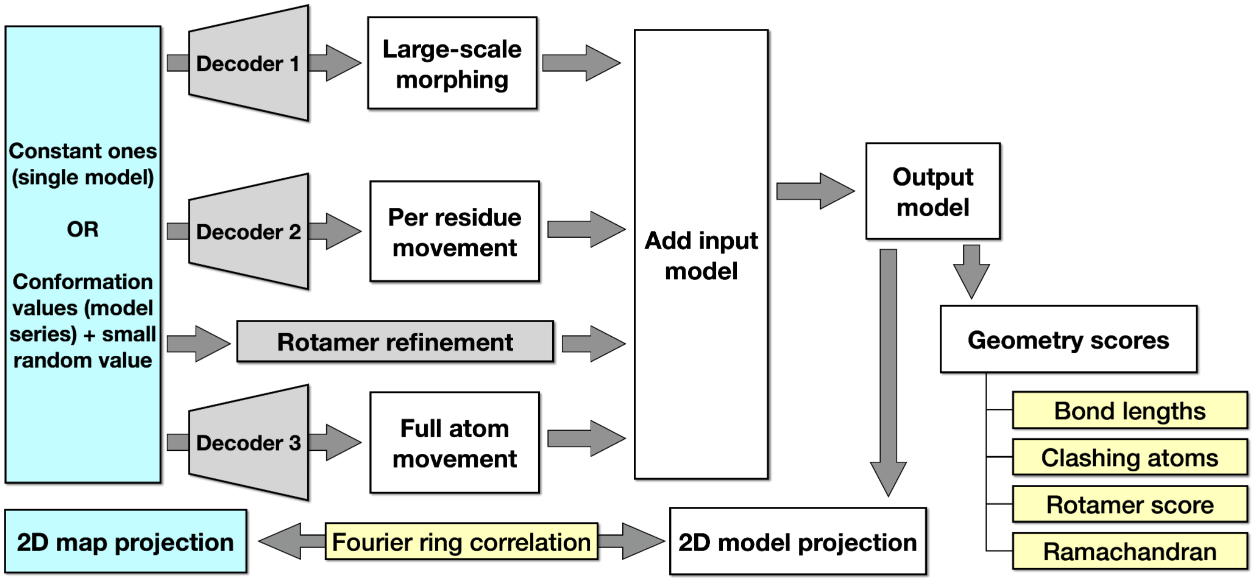

Figure S1. Workflow diagram for molecular model refinement protocol.

bioRxiv preprint doi: https://doi.org/10.1101/2024.09.27.615511; this version posted September 27, 2024. The copyright holder for this preprint (which was not certified by peer review) is the author/funder, who has granted bioRxiv a license to display the preprint in perpetuity. It is made available under aCC-BY 4.0 International license.

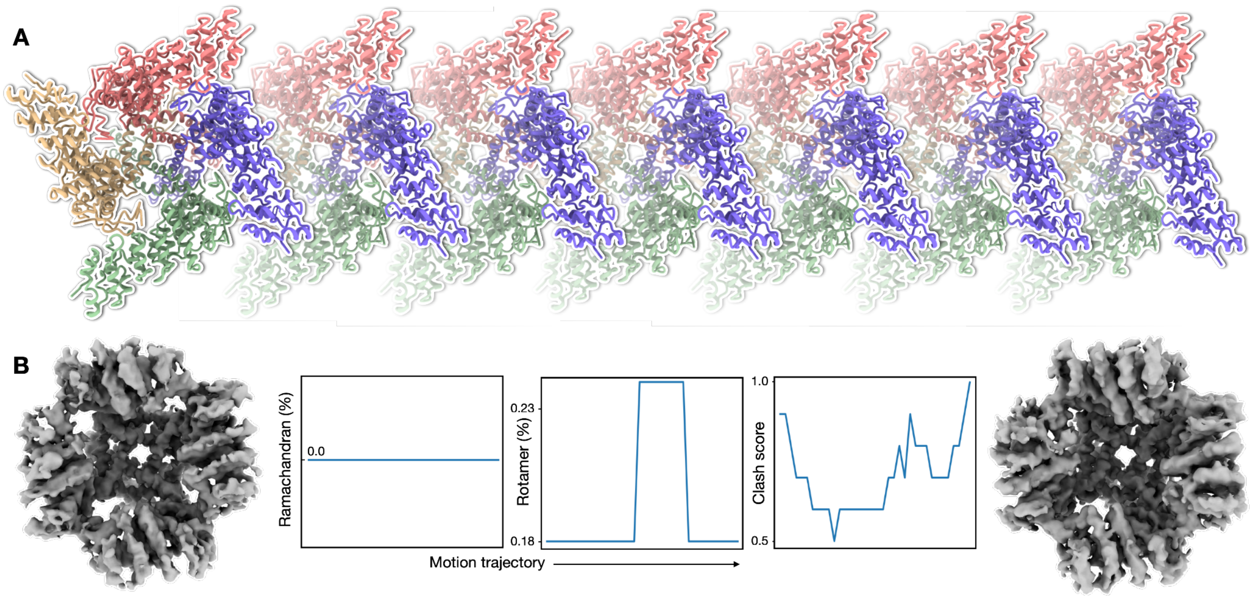

Figure S2. Model series refinement of TRPV1. **(A)** Snapshots of GMM refined molecular model series along one continuous motion trajectory of TRPV1 from dataset EMPIAR-10059. **(B)** Left and right panels - 3D reconstruction of the first and last frame of the motion; middle three panels - Ramachandran, rotamer outlier, and clash score for models along the motion trajectory.

bioRxiv preprint doi: https://doi.org/10.1101/2024.09.27.615511; this version posted September 27, 2024. The copyright holder for this preprint (which was not certified by peer review) is the author/funder, who has granted bioRxiv a license to display the preprint in perpetuity. It is made available under aCC-BY 4.0 International license.

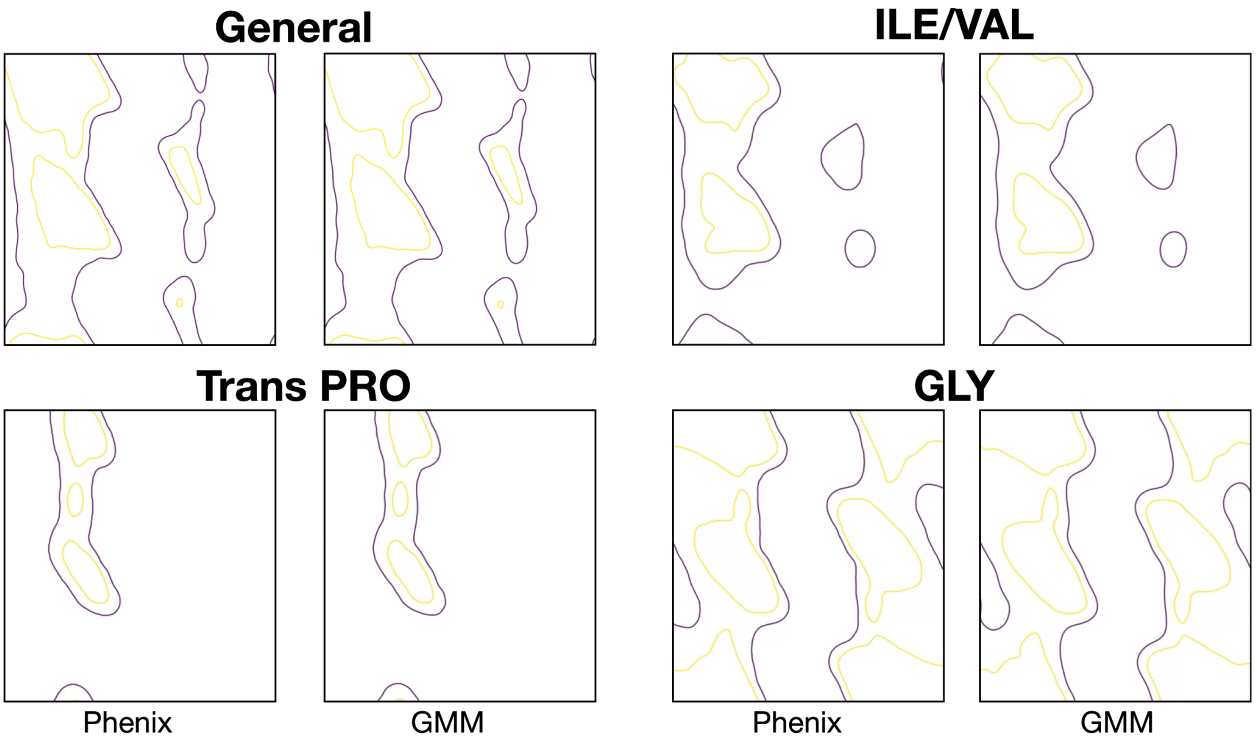

Figure S3. Comparison between histogram representation of the Ramachandran plot from Phenix, and the GMM representation of the corresponding plot. Four types of Ramachandran plots are shown, and the contour lines indicate the boundary of allowed (yellow, 2%) and outlier (purple, 0.05%). The Phenix and GMM plots of the same type should be nearly identical.

bioRxiv preprint doi: https://doi.org/10.1101/2024.09.27.615511; this version posted September 27, 2024. The copyright holder for this preprint (which was not certified by peer review) is the author/funder, who has granted bioRxiv a license to display the preprint in perpetuity. It is made available under aCC-BY 4.0 International license.

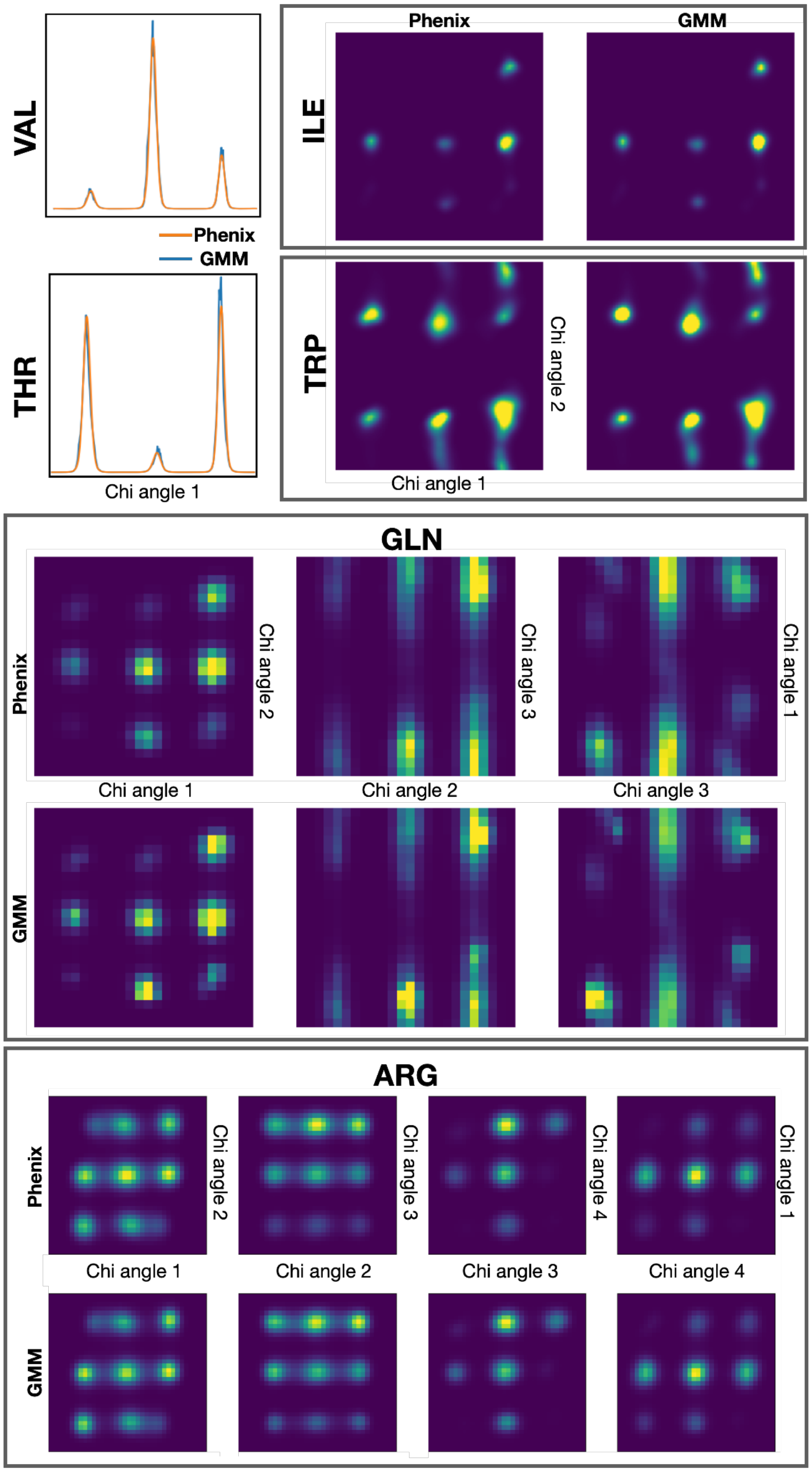

Figure S4. Comparison between histogram representation of the preferred rotamer from Phenix, and the GMM representation of the corresponding plot. Here we show two types of sidechains with one Chi angle (VAL, THR), two types with two Chi angles (ILE, TRP), one type with three Chi angles (GLN), and one with four Chi angles (ARG).

bioRxiv preprint doi: https://doi.org/10.1101/2024.09.27.615511; this version posted September 27, 2024. The copyright holder for this preprint (which was not certified by peer review) is the author/funder, who has granted bioRxiv a license to display the preprint in perpetuity. It is made available under aCC-BY 4.0 International license.

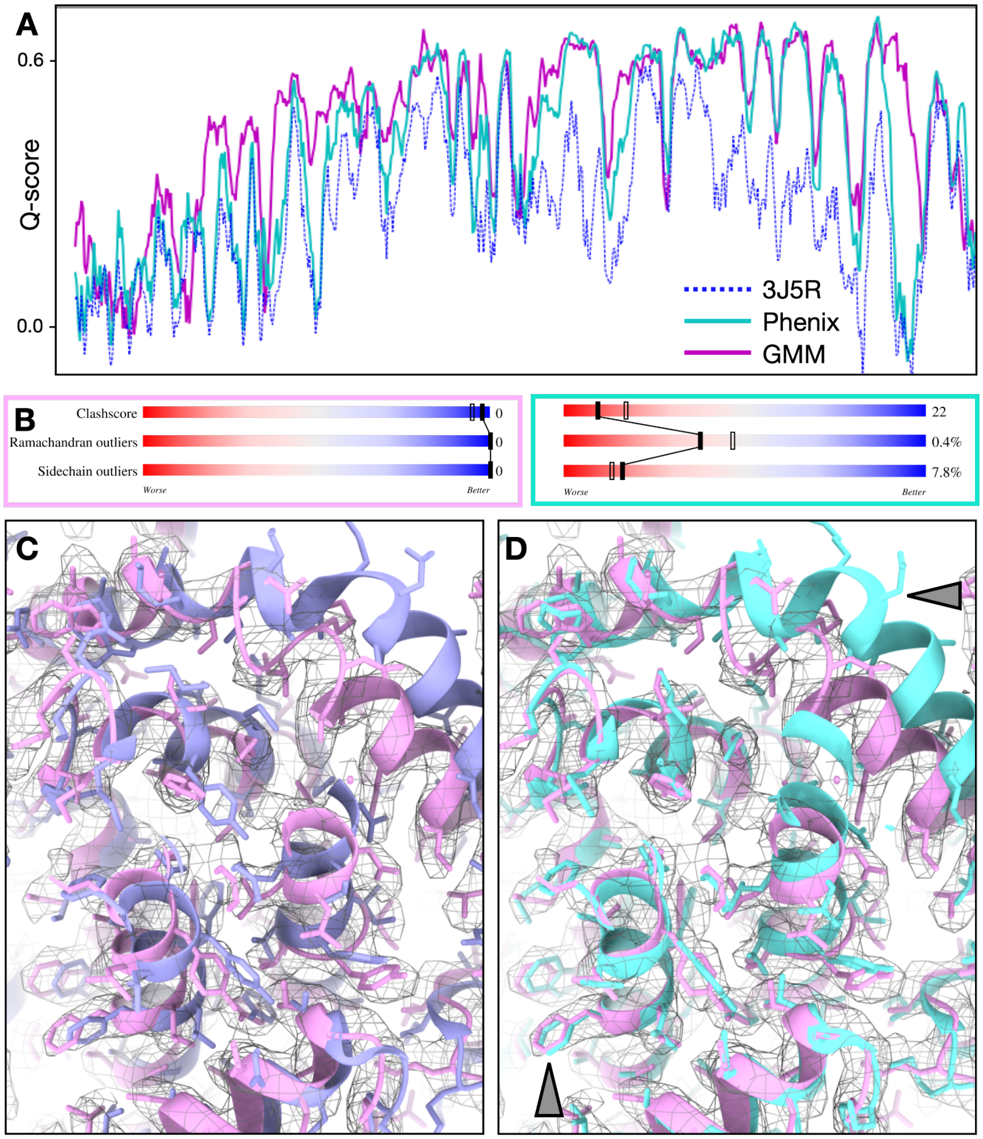

Figure S5. Comparison between the GMM-based model refinement and Phenix real-space refinement in the TRPV1 example shown in Figure 1. **(A)** Q-score comparison. Blue - original PDB model; cyan - Phenix real space refinement; pink - GMM-based refinement. **(B)** PDB validation metric comparison. Left - GMM-based refinement; right - Phenix real-space refinement. **(C)** Overlay of the original (blue) and GMM refined (pink) model with the CryoEM map. **(D)** Overlay of the original (blue) and Phenix refined (cyan) model with the CryoEM map.

bioRxiv preprint doi: https://doi.org/10.1101/2024.09.27.615511; this version posted September 27, 2024. The copyright holder for this preprint (which was not certified by peer review) is the author/funder, who has granted bioRxiv a license to display the preprint in perpetuity. It is made available under aCC-BY 4.0 International license.

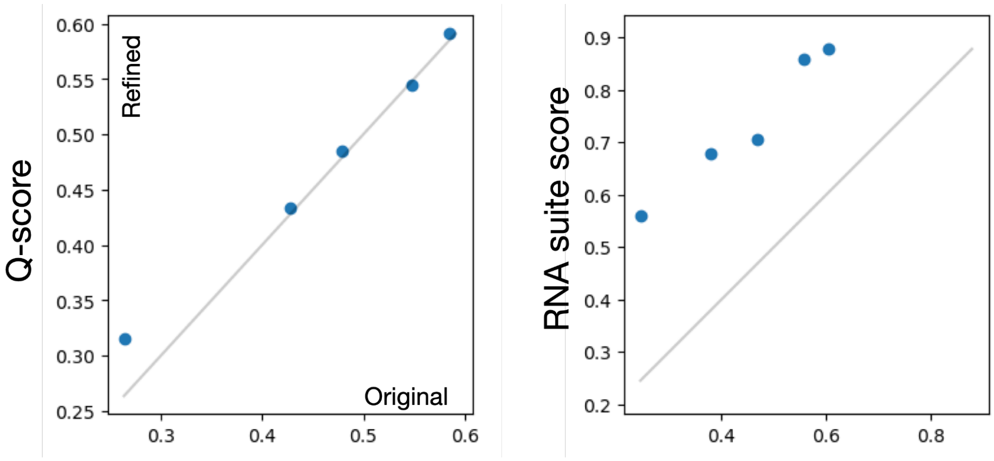

Figure S6. Comparison of the original and refined version of 5 models with RNA from the PDB. Left - Q-score; right - RNA suite score (higher score indicates better RNA backbone geometry).

bioRxiv preprint doi: https://doi.org/10.1101/2024.09.27.615511; this version posted September 27, 2024. The copyright holder for this preprint (which was not certified by peer review) is the author/funder, who has granted bioRxiv a license to display the preprint in perpetuity. It is made available under aCC-BY 4.0 International license.

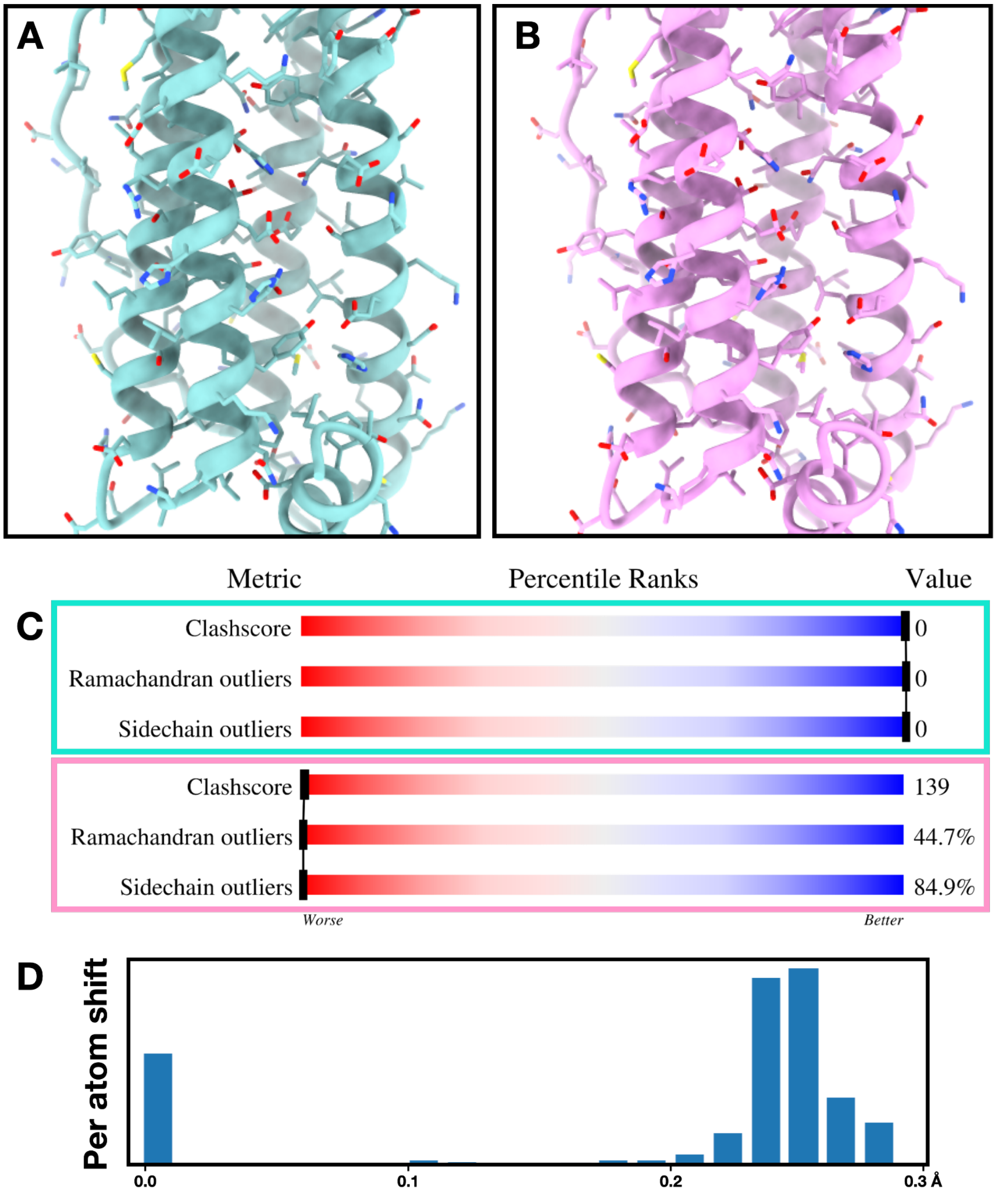

Figure S7. Sensitivity of the PDB validation metrics. (A) Input model of apoferritin (PDB-8T4Q). (B) Output model from the GMM-based refinement with an inverse loss function, using the model from A as input. (C) Comparison of PDB validation scores between the input (top) and inverse "refined" model (bottom). (D) Histogram of per-atom shift distance between the two models.

bioRxiv preprint doi: https://doi.org/10.1101/2024.09.27.615511; this version posted September 27, 2024. The copyright holder for this preprint (which was not certified by peer review) is the author/funder, who has granted bioRxiv a license to display the preprint in perpetuity. It is made available under aCC-BY 4.0 International license.

Supplementary video 1. Model series generated from the TRPV1 dataset (EMPIAR-10059), along a linear trajectory in the conformational space.

Supplementary video 2. Model series generated from the spliceosome dataset (EMPIAR-10180), along a linear trajectory in the conformational space.

Supplementary video 3. Model series generated from the spliceosome dataset (EMPIAR-10180), along a circular trajectory in the conformational space.
